# Round the Hospitals

**Published:** 1919-12-20

**Authors:** 


### ROUND THE HOSPITALS.

On December 10 the King held an Investiture in the ball-room of Buckingham Palace and personally conferred decorations on the following members of the nursing services:—The Royal Red Cross, 1st Class: Miss Mary Bishop, Mrs Gertrude Green, and Miss Bessie Rankin, Q.A.I.M.N.S. (R.). Miss Grace Craig, Miss Marion Thomas, and Miss Madeline Watts, T.F.N.S. The Royal Red Cross 2nd Class: Miss Minnie Maclean and Miss Christina Macrae, Q.A.I.M.N.S., Miss Nina Cairns, Miss Mary Clery, Miss Lynda Coates, Miss Estelle Doyle, Miss Mary Potts, Miss Amelia Pressley, Miss Beatrice Reid, Miss Violet Riley, Miss Mary Ross, and Miss Maude Todman, Q.A.I.M.N.S. (R.); Miss Louisa Berry, Miss Edith Moore, Miss Elizabeth Neil, Miss Florence Pierrepont, Miss Emma Stokes,

**ROUND THE HOSPITALS**—*(continued).*

and Miss Edith Willis, T.F.N.S.; Miss Jenny Morris and Miss Hilda Palmer, Civil N.S.; Miss Beatrice Beeson and Miss Mabel Winch, Civil and War Hospitals; Miss Amy Wilson, B.R.C.S.; Miss Gladys Gardiner, Miss Lily Privett, Mrs. Mary Stein, and Mrs. Jessie Townsend-Whitling, V.A.D. The Military Medal was conferred on Miss Susan Munroe, Q.A.I.M.N.S. (R.). All the above ladies with Dame Sidney Browne, Matron-in-Chief T.F.N.S., were received by Queen Alexandra at Marlborough House subsequent to the investiture.

THE KING held a second Investiture on December 11, in the Ballroom of Buckingham Palace, and personally decorated members of the Nursing Services as follows:—The Royal Red Cross, First Class: Miss Marion Branson, Miss Olive Stinton, and Miss Lucy Toller (who also received the Military Medal), Q.A.I.M.N.S.; Miss Grace Corder, Annie Mrs. Nash, and Miss Jeanie Strachan, Q.A.I.M.N.S. (R.). The Royal Red Cross, Second Class: Miss Mabel Chester-Webb, Q.A.R.N.N.S., Miss Clarke Williams, Q.A.I.M.N.S., Miss Helen Caig, Miss Agnes Cummings, Miss Margaret Macdonald, Miss Mary McDonald, Miss Lily Millar, Miss Florence Puddicombe, Miss Alice Rogers, Miss Amy Russell, Miss Ino Skinner, and Miss Hilda Starbuck, Q.A.I.M.N.S. (R.); Miss Rosina Bowyer, Miss Jean Halliday, Miss Beatrice Matthews, Miss Margaret McIntyre, Miss Eliza Nicol, Miss Helen Romer, Margaret Mrs. Rose, Miss Sophie Thomson, Miss Dorothy Ward, Miss Florence Widdop, and Miss Annie Wood, T.F.N.S.; The Dowager Countess of Suffolk, B.R.C.S.; Mrs. May Price, East African N.S.; Miss Gertrude Howell-Evans, Miss Caroline Kernan, Miss Emma Masters, Miss Mabelle Milnes, Miss Geraldine Platt, Miss Marjorie Platt, and Miss Katherine Ryott, V.A.D. His Majesty conferred the Military Medal on Miss Maud Abraham, Q.A.I.M.N.S.(R.) All these ladies, together with Miss A. B. Smith, Matron-in-Chief Q.A.I.M.N.S., were subsequently received at Marlborough House by Queen Alexandra.

THE final list of war decorations contains the names of many members of the Nursing Services. *For Service in France.*—Royal Red Cross, Second Class: Sister G. S. Surman, Sister G. Youatt, and Miss G. Bell, V.A.D., all of Queen Alexandra Hospital, Dunkirk. *For Service in Italy.*—Royal Red Cross, First Class: Mrs. McEwen, Sister-in-Charge, Q.A.I.M.N.S. (R.). Second Class: Sister W. Johnson, T.F.N.S., Sister-in-Charge N. A. Maling, Q.A.I.M.N.S. (R.). *For Service in the Balkans and Army of the Black Sea.*—Royal Red Cross, First Class: Asst. Matron L. E. Barrow, A.R.R.C., Asst. Matron E. I. E. Jones, Sister-in-Charge M. Wright, Q.A.I.M.N.S. (R.). Second Class: Sister E. K. Broughwyk, Sister H. L. Carpenter, Sister S. R. Finlay, Sister B. G. Graham, Sister M. C. Mann, Sister M. Symington, Staff Nurse M. M. Walker, Q.A.I.M.N.S. (R.): Staff Nurse A. Dobson, Sister H. M. Hamilton, Staff Nurse M. E. G. Jennings, T.F.N.S. *For Service in East Africa.*—Royal Red Cross, Second Class: Matron A. M. Muggeridge. *For Service in India.*—Royal Red Cross, Second Class: Senior Nursing Sister E. G. Browne, Sister M. E. I. Hornsby, Sister U. Steel, Sister Q. T. A. White. Other awards will be given in our next issue.

THE Nurses' Registration (No. 2) Bill, which was passed through the House of Commons, was read a second time in the House of Lords on the 15th inst. The Nurses' Registration (Ireland) Bill and the Scotch Bill on the same subject were also read a second time in the Lords.

WE hope to hear soon that a South African Branch of the College of Nursing has come into being. A *rapprochement* with the South African Trained Nurses Association has been brought about, and the General Secretary, Miss Alexander, at a meeting held on October 17 in the Nurses' Home of the Kimberley Hospital, advised the nurses in the course of an interesting address to become members of the College. She spoke of the great strides that the College of Nursing was taking to better the profession, and urged that now was the time to co-operate with England as the South African Military Nursing Service had established a very good name during the war. We believe that co-operation and unity throughout the Empire will bring a great accession of strength to the nursing profession. We found in the war we had something to teach and something to learn from our comrades overseas, and should be exceedingly glad to draw the bonds of kinship closer.

V.A.D. CANDIDATES who have completed two years' service in properly equipped military hospitals are to be eligible to serve in the children's hospitals of the Metropolitan Asylums Board under the following agreement: They must undertake to stay for two years, but they will be allowed to sit for the two-years' certificate at the end of their first year, and for the three-years' certificate at the end of their second year. A footnote will be added to their certificate to the effect that one year of training has been excused in consideration of two years' previous work at military hospitals. The commencing salary is to be that of a second-year probationer. The fact that one day's leave in seven and a forty-eight hours' week are now in force in all the M.A.B. institutions makes this offer one to be grasped at.

THE present acute shortage of nurses is being felt to a distressing extent in Bristol, where patients are being constantly refused because of the inadequacy of the staff to deal with more cases. A sub-committee of the Bristol Health Committee was recently appointed to examine into the causes of the shortage and a detailed report has been presented by Mr. H. J. Maggs embodying their recommendations. They urge that salaries and wages should be increased all

**ROUND THE HOSPITALS**—(*continued*).

round, that the 10 per cent. bonus should be permanently added, that hours should be reduced and conditions of service generally improved. It was stated that while every hospital was short of nurses there were 691 women out of employment in Bristol. The tide of fashion in the families from which probationers have normally been drawn has for the moment evidently turned strongly against nursing. It is absolutely necessary not only to improve the conditions of service but to give this improvement wide publicity. An intelligent campaign must be set on foot to disseminate true information about the lives of nurses, for we fear there has been much misrepresentation and that a strong and unreasonable prejudice against adopting the profession is gaining ground. Bristol has more than one institution where the conditions of service for the nurses are all that could be desired.

---

An appeal from Miss Turner, the matron, which appears in the Hampshire papers, for help in keeping Christmas at the County Hospital, is a useful reminder that the onus of making festival for the patients at this season ought rightly speaking to devolve on the public, rather than on the staff. Where the occasion is made the medium whereby additional interest and voluntary help are secured for the hospital, the effects of Christmas are felt throughout the whole year and blossom into many good deeds. The things in which we are really interested, for which we delight to give and get money, are those in which we take an actual, if humble, share. Almost any kind of personal service can be turned to good account at Christmastime, and a great blunder is committed by managers when they leave the hard-pressed matron and sisters to wrestle unaided with the task of ward decoration, presents to patients, and entertainments. Every single person enlisted in the cause and set to work is a radiating source of energy, and hence of financial support to the hospital.

---

The *Nurses' League Annual*, edited by the Rev. A. Lombardini, and published by the Kensington Infirmary, is a capital number this Christmas, and will interest members of the nursing profession far and near. Field-Marshal Earl Haig sends a special message of thanks to the brave devoted women who served their country as nurses during the Great War. "They did more," he goes on to say, "than bring comfort and happiness to the wounded and sick. They contributed directly to the maintenance of the strength of our Armies in the field," and "helped directly to secure the victory of our cause." Sir Arthur Stanley also sends a message wishing he could get all the nurses in this country together so that he could tell them what is in the minds of those who founded the College of Nursing, and touching on the ways in which high ideals can be linked with a general improvement in the material conditions and economic status of nurses. The Editor's own plea for a Nurses' War Memorial and his suggestion as to the form it should take will be read with great interest by all nurses. Other good fare is provided for the reader, and the lighter side is not omitted. The illustrations are particularly good. Copies of the *Journal* can be obtained from its Editor, price 1s. 3d. post free.

---

The doll show at the Mansion House in aid of the funds of St. Bartholomew's Hospital proved such a phenomenal success that it is marked out for imitation. Every stall was eventually sold out, for the Lady Mayoress bought those which remained over to give to children in the East End hospitals. The dolls were all dressed by lady assistants in the leading West-End drapery establishments, with the exception of two fine specimens dressed by ex-sailor and ex-soldier employees. The utmost ingenuity and good workmanship were displayed in designing and carrying out the details of the costumes, and the prices realised proved once again, if anyone doubted it, that the public has plenty of money to spend when its palate is tickled. The hospitals, as a rule, strangely ignore the readiness of business women to devote their skill and time most generously to further the interests of the sick poor.

---

The recent prize-giving at the East Suffolk and Ipswich Hospital brought to notice a great advance in the curriculum of the training-school. The matron, Miss Deane, alluded in her address at the meeting to the inclusion of massage and remedial Swedish exercises in the course, and mentioned that all the massage candidates had passed the examination of the I.S.T.M. in London. Dr. Moseley told them that the hospital is entering upon a new era; new departments are being formed, medical cases are separated entirely from surgical cases, and specialisation is taking place in all branches of the work. Thus the nurses have the opportunity of acquiring a really sound and scientific training in their work. The Chairman, Mr. Cobbold, has constantly shown his regard for the nurses in generous and practical ways, and has done his utmost to secure them adequate salaries and plenty of off-duty time and holidays. The gold medal was won by Nurse Phyllis L. Murray, and the silver medal by Nurse Dorothy Arden, who also carried off the matron's special prize.

---

A dinner of the staff (medical officers, sisters, and V.A.D.s) of No. 14 General Hospital (Wimereux) will be held in London, at 8 P.M., on Monday, February 2, with Lieut.-General Sir John Goodwin in the chair. Day dress will be worn. Application for tickets (12s. inclusive) should be made, together with remittance, to Miss Lawley, 9 Seymour Street, London, W. 1, when the address of the restaurant will be given.

---

The work done for some years past by the Scottish Board of Health in the examination and certification of trained sick and fever nurses is worthy to be held in remembrance as the precursor of State examinations. The last examination was held at Glasgow, Edinburgh, Dundee, and Aberdeen, and 228 nurses presented themselves. The subjects

**ROUND THE HOSPITALS**—(*continued*).

were Elementary Anatomy and Physiology; Hygiene and Dietetics; Medical and Surgical Nursing; Midwifery (for Poor-Law Trained Nurses) and Infectious Diseases (for fever-trained nurses only). The experience gained in the course of these examinations cannot fail to be of service to the Nursing Registration Council. The Scottish Board has acted as a pioneer in this direction, and has done for Scotland what has not been attempted on a similar scale south of the Tweed—it has accustomed nurses to the idea of undergoing a public test of their nursing qualifications.

The Newcastle Poor-law Infirmary, which can boast of being the largest in size between Leeds and Edinburgh, was *en fête* on November 26, when Lord and Lady Armstrong attended to present prizes and certificates to nurses successful in the examinations. Mrs. Snowdon, Matron of the Infirmary, gave her guests tea after the presentation. The Chairman of the Infirmary Committee recorded with pride that the Infirmary nurses had served with credit in every theatre of war, and forecasted changes for the better in the matter of duty hours. Nine nurses received their training certificates, of whom Nurses E. Lowerson, E. P. Phillips, and S. T. Lawson were accorded the Heath prizes. These nurses also received, in the order given, first, second, and third prizes from the Guardians.

The figures presented at the annual meeting of the Huddersfield and District Victoria Nurses' Association show that each visit of the nurses now costs 1s. 1½d., as against 7d. before the war. In other words, the cost has about doubled. The Committee started their year with an adverse balance at the bank of £180, but have worked so well that they closed it with a balance to the good of £195. The principal need in the future is for a separate home for the maternity nurses, and once started it is estimated it would be self-supporting owing to the demand for training in midwifery. Miss Ross, the Queen's Institute Inspector for the North-East Midlands, paid a high compliment to Huddersfield as a pioneer in all work relating to child welfare. Much more money is needed if their full programme is to be carried on, but no doubt it will be forthcoming.

We desire to call attention to a little hospital in Scotland, the Dundee Women's Hospital, with a daily average of nineteen occupied beds, because the managers appear to have solved the problem of economy in a remarkably successful manner. Including staff, the household averages twenty-eight persons, and these were provided for during the past year at an expenditure of £565 15s. At the recent annual meeting Dr. Emily Thomson, who has been connected with the institution since it was opened, and whose approaching marriage was announced, called attention to the fact that this sum worked out at less than 10s. a week. She could testify from personal observation that there was no underfeeding or discontent. We should be very glad to have details of this marvellous housekeeping feat.

The Cardiff Councillors will shortly take over their mental hospital at Whitworth from the military, and a general rearrangement of the medical and nursing system in force has become necessary. Under the War Office the hospital was staffed by certificated women nurses, and one of the councillors contended that the Committee should return to their old system of male attendants. Lieut.-Colonel Goodall informed the councillors that 2,000 acute mental cases had been nursed by women, and stated that nursing by women was by far the most enlightened system and was best for the patients. It was agreed that the female nursing staff should be retained, with due regard to the interests of the male staff, some of whom have been fifteen years at the hospital.

At Ramsgate the war memorial is to take the form of a large children's annexe, containing 40 beds, to the general hospital. The unrivalled air greatly facilitates the treatment of all children's diseases. Chronic cases are dealt with in many institutions in Thanet, but much more accommodation is needed for ordinary medical and surgical cases among children. An effort should be made at the same time to raise the number of beds, if possible, to 100, so that the nurses may not find themselves excluded from registration. The difficulty of securing probationers will be immeasurably increased for matrons of institutions not reckoned as training-schools when the Act comes into force.

The New South Wales Bush Nursing Association, of which we have received the report, struggled on bravely through the last year of war, and kept most of its centres going in spite of the immense call for nurses in other directions. At least six new centres are waiting for nurses before they can be affiliated. An interesting example of the value of the nurse to settlers is reported from Lightning Ridge, where pneumonic influenza showed itself. The nurse promptly reported the first suspicious cases to her committee, who lost no time, in conjunction with the medical authorities, in isolating the cases and the contacts. There were fourteen cases, all of which eventually recovered, and a severe epidemic was averted. Contrasted with what took place in this and other countries the episode points to admirable organisation.

Best congratulations to Miss Lily Dickinson, the nurse who was awarded £1,394 at the Manchester Assizes for professional services to the late Mr. Thomas Guest. The patient deferred payment to his nurse during his lifetime under the curious plea that his insurance made him richer dead than alive, and she was able to show that he had promised to leave her an annuity of 30s. a week.

